# Symptoms in primary care with time to diagnosis of brain tumours

**DOI:** 10.1093/fampra/cmx139

**Published:** 2018-02-06

**Authors:** Mio Ozawa, Paul M Brennan, Karolis Zienius, Kathreena M Kurian, William Hollingworth, David Weller, Willie Hamilton, Robin Grant, Yoav Ben-Shlomo

**Affiliations:** 1 Population Health Sciences, Bristol Medical School, University of Bristol, Bristol, UK; 2 Translational Neurosurgery Unit, Centre for Clinical Brain Sciences, University of Edinburgh, Edinburgh, UK; 3 Brain Tumour Research Group, University of Bristol, Institute of Clinical Neuroscience, Learning and Research Building, Southmead Hospital, Bristol, UK; 4 Centre for Clinical Brain Sciences, University of Edinburgh, Edinburgh, UK; 5 Institute of Clinical Neurosciences, University of Bristol, Southmead Hospital, Bristol, UK; 6 Centre for Population Health Sciences, University of Edinburgh, Edinburgh, UK; 7 Primary Care Diagnostics, University of Exeter Medical School, College House, St Luke’s Campus, University of Exeter, Exeter, UK

**Keywords:** Brain tumour, delay in accessing care, diagnosis, National Audit of Cancer Diagnosis in Primary Care, symptoms

## Abstract

**Background:**

Brain tumours often present with varied, non-specific features with other diagnoses usually being more likely.

**Objective:**

To examine how different symptoms and patient demographics predict variations in time to brain tumour diagnosis.

**Methods:**

We conducted a secondary analysis of brain tumour cases from National Audit of Cancer Diagnosis in Primary Care. We grouped neurological symptoms into six domains (headache, behavioural/cognitive change, focal neurology, ‘fits, faints or falls’, non-specific neurological, and other/non-specific) and calculated times for patient presentation, GP referral, specialist consultation and total pathway interval. We calculated odds ratios (ORs) for symptom domains comparing the slowest to other quartiles.

**Results:**

Data were available for 226 cases. Median (interquartile range) time for the total pathway interval was 24 days (7–65 days). The most common presentation was focal neurology (33.2%) followed by ‘fits, faints or falls’ and headache (both 20.8%). Headache only (OR = 4.11, 95% CI = 1.10, 15.5) and memory complaints (OR = 4.82, 95% CI = 1.15, 20.1) were associated with slower total pathway compared to ‘fits, faints or falls’. GPs were more likely to consider that there had been avoidable delays in referring patients with headache only (OR = 4.17, 95% CI = 1.14, 15.3).

**Conclusion:**

Patients presenting to primary care with headache only or with memory complaints remain problematic with potentially avoidable delays in referral leading to a longer patient pathway. This may or may not impact on the efficacy and morbidity of therapies. Additional aids are required to help doctors differentiate when to refer headaches and memory complaints urgently for a specialist opinion.

## Introduction

The incidence of brain tumours is low; age-adjusted incidence rates for all gliomas range from 4.7 to 5.7 per 100000 persons (1). This means that the diagnosis of brain tumour is very rare in primary care populations. While the diagnosis of cancer is usually made in secondary care, most patients will have seen their GP prior to a diagnosis (2–4). Further, patients can present with a wide range of different symptoms which may be common (e.g. headache), non-threatening or may be thought of as part of a normal ageing process (e.g. memory loss). The non-specificity of these symptoms creates a diagnostic challenge for all clinical staff. Current guidelines in the UK recommend that all patients with suspected CNS tumour must be seen by a specialist within 2 weeks of referral by their GP; despite the introduction of this guideline in 2005, there appears to have been little improvement in the diagnostic interval (5) (the time from first presentation with symptoms to diagnosis) over the last decade (6). Indeed, most recent figures show only 1% of cases with suspected brain tumour are diagnosed through the ‘suspected cancer’ 2-week wait process, while 17% are GP referrals through usual pathways, and 58% are diagnosed after an accident and emergency attendance (7).

Several studies have examined case series of patients with brain tumours and have quantified the frequency of the most common presenting symptoms (8,9); in some cases deriving predictive values by comparing this to age–sex matched control patients in primary care (10–15). A systematic review (16) found that all symptoms had in general low positive predictive values for brain tumours, apart from new-onset epilepsy. Few studies have investigated how symptoms may influence the time to diagnosis. The National Audit of Cancer Diagnosis in Primary Care (NACDPC) has previously found that around a third (35.2%) of patients with brain tumours took 15 days or more to present to their GP (17) and 21.4% of cases required three or more consultations before referral compared to 17.9% for all cancers or as little as 2.9% for breast cancer patients (18).

This study examines whether different clinical presentations are associated with variations in the patient pathway to diagnosis and where future interventions could be best targeted to reduce diagnostic delay and possibly improve patient prognosis.

## Material and methods

### Data

We analysed data from the (English) NACDPC (2009–2010). Data were collected from 18879 patients by 1170 practices (~14% of all practices in England) in 20 cancer networks using an audit template and information from their practice clinical records and hospital correspondence. Any screen-detected or incidental cancers were excluded from the audit. Patient demographics and the information related to the assessment process in primary care were collected (for full details concerning the NACDPC methods, see the report by Royal College of General Practitioners) (19).

### Outcomes

**Figure 1.**
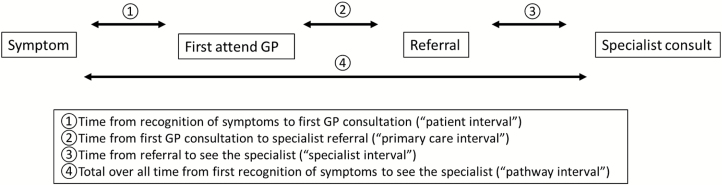
The pathway and time to diagnosis for patients with brain tumour

Only patients with a confirmed diagnosis of brain tumour (no details on specific pathology were available) were selected for this analysis. We examined time to four specific outcomes to try and understand the clinical pathway from symptom onset to specialist consultation (see Figure 1 for visual representation): (i) time from patient recognition of symptoms until first GP consultation (‘patient interval’); (ii) time from first GP consultation until referral to specialist (‘primary care interval’); (iii) time from referral until specialist attendance (‘specialist interval’); (iv) total time from patient recognition of symptoms until first specialist visit (sum of 1 and 2 and 3 above) (‘pathway interval’).

In addition, we looked at three other related outcomes that may indicate a suboptimal referral interval: (i) if the patient attended primary care three or more times before referral and (ii) the GP’s response to the following questions (a) ‘Would rapid access to investigations have altered your management of this case?’ and (b) ‘Were there any avoidable delays to this patient’s journey?’ In this latter case, GPs could respond ‘No’, ‘Yes’ or ‘Unsure’. Due to small numbers, we combined ‘Unsure’ and ‘No’ to create a binary outcome variable (‘Yes’ versus ’Unsure/No’). These last two variables are retrospective in nature.

### Clinical symptoms

The information on patient records was collected by GPs or primary care professionals. We grouped individual symptoms into six domains based on categorizations of previous papers and the region of brain likely to be causing the symptom: intracerebral damage—focal neurology; intracerebral damage—cognitive/behavioural; intracerebral excitation (seizure); intracranial extracerebral damage (cranial nerve); raised pressure (headache); and ‘non specific’ based on specialist opinions (PB, KZ, RG) (see supplementary table 1). We created the following domains: (i) headache; (ii) behavioural/cognitive change; (iii) focal neurology including stroke; (iv) episodic attacks—‘fits, faints and falls’; (v) non-specific neurological; and (vi) other/non-specific features. Headache and behavioural/ cognitive change were further divided into two subgroups: headache was divided into headache only and headache plus additional features recorded, whilst behavioural/cognitive change was divided into confusion and memory only subgroups. If more than one symptom was recorded (other than for the headache plus group), we chose what we considered to be the main symptom for classification purposes.

### Other covariates

We also examined the following covariates: gender, age group (<60, 60–70, >70 years), ethnicity (white British versus other), whether the patient had problems in communication, was housebound, whether the GP ordered investigations before referral, type of referral, where patients first presented, and which specialist was chosen for the referral.

### Statistical analysis

We calculated the median (interquartile range) time to diagnosis (in days) according to patient, referral, specialist and pathway intervals by symptom domains, and other factors. As the time data were highly skewed, we derived a binary outcome variable indicating slower time interval by deriving quartiles and comparing patients in the slowest versus the other three quartiles. We compared each symptom domain relative to fits, faints and falls (baseline group) as this domain was associated with the shortest pathway interval.

We calculated odds ratios (ORs), 95% confidence intervals (CI) and *P* values using multivariable logistic regression and treating symptom domain as a dummy variable. We calculated crude and multivariable ORs, adjusting for age group, sex and ethnicity as these covariates are potential confounders as they may determine how symptoms are perceived by the patients and present to the GP as well as influencing the time to see GP and referral. Because of missing data for the time intervals, we undertook multiple imputation using chained equations for our binary outcomes so that we could use all the cases in our logistic regression model. This analysis is potentially less biased due to missing data. The imputation model included all variables from our analysis model as well as covariates shown in Table 1. We used 20 cycles for the chained equations and derived 10 imputed datasets, which were then combined using Rubin’s rules to derive the appropriate ORs, 95% CI and *P* values using the mi estimate command in Stata. The multivariable ORs are based on the imputed dataset to maximise statistical power, given the relatively small sample size.

**Table 1.** Sociodemographic characteristics of patients of brain tumour, *n* = 226

Variable	Frequency	%		Frequency	%
Age group			Investigation before referral		
<60	82	36.3	No	142	62.8
60–69	55	24.3	Yes	70	31.0
≥70	84	37.2	Unknown	14	6.2
Unknown	5	2.2			
			Which specialist to be referred		
Sex			Neurology	55	24.3
Female	105	46.5	Accident and emergency	18	8.0
Male	121	53.5	Medicine and geriatrics	50	22.1
			Ophthalmology	17	7.5
Ethnicity			Neurosurgery	19	8.4
White British	177	78.3	Paediatrics	11	4.9
Other	29	12.8	Stroke	9	4.0
Unknown	20	8.9	Miscellaneous	17	7.5
			Unknown	30	13.3
Housebound					
No	187	82.7	Type of referral		
Yes	27	12.0	Emergency	90	39.82
Unknown	12	5.3	Not referred by practice	34	15.04
			2 week/private	41	18.14
Problems in communication			Routine	39	17.26
No	187	82.7	Unknown	22	9.73
Dementia	4	1.8			
Language barrier	8	3.5	Attended 3+ before referral		
Leaning difficulty	2	0.9	No	119	62.7
Mental Health	2	0.9	Yes	76	33.6
Poor vision	1	0.4	Unknown	31	13.7
Speech impediment	11	4.9			
Unknown	11	4.9	Rapid access investigations		
			No	157	69.5
Symptoms			Yes	46	20.4
Headache	47	20.8	Unknown	23	10.1
Headache only	16				
Headache plus	31		Avoidable delays in patient journey		
Behavioural/cognitive	28	12.4	No	153	67.7
Confusion	14		Yes	68	30.1
Memory	14		Unknown	5	2.2
Focal neurology	75	33.2			
Fits, faints or falls	47	20.8			
Non-specific neurological	11	4.9			
Other/non-specific	18	8.0			

## Results

There were 226 patients (96.6%) with information on presenting symptoms from 234 brain tumour cases. The age distribution was bimodal (younger and older) with roughly equal numbers of men and women (Table 1) The most common symptom domain was focal neurology including stroke (33.2%), followed by episodic attacks—‘fits, faints or falls’ (20.8%) and headache (20.8%). About 30% of cases had experienced three or more consultations prior to referral. In around one-third of the cases GPs considered, or were not sure if, there had been avoidable delays. In around 20% the GPs felt that rapid access to investigations would have been helpful.

The median (interquartile range) of the pathway interval was 24 days (7–65 days) (Table 2). Younger patients (< 60 years) had longer delays on the pathway. There were marked variations in the pathway interval by symptom domain. The shortest time was seen for episodic attacks – ‘fits, faints or falls’ (10 days) whilst the longest interval was seen for memory loss (62 days). Patients who had investigations before referral to specialist care had a longer pathway interval.

**Table 2.** Median and interquartile range of time to diagnosis (days) by sociodemographic characteristics of patients with a brain tumour

Variable	Time to diagnosis							
	Patient interval^a^		Primary care interval^b^		Specialist interval^c^		Pathway interval^d^	
	Media *n*	Interquartile range	Media *n*	Interquartile range	Media *n*	Interquartile range	Media *n*	Interquartile range
Age group								
<60	5.5	0–25.5	3	0–31.5	6	0–19	25.5	8–81
60–69	8	1–26	2	0–6	2	0–12.5	20.5	7–63
>60	5	0–15.5	1	0–15	7	0–24	22	7–54
Sex								
Female	5	0–21	1.5	0–13.5	4	0–15	25	7–60.5
Male	8	1–26.5	2	0–15	6	0–17	24	8–66
Ethnicity								
White British	6	0–22	2	0–16	4	0–15	24	7–66
Other	6	0–23.5	0	0–7	5	0–25	20	7–60
Housebound								
No	7	0–29	1	0–11	6	0–17	25	7–77
Yes	3	0–8	4	0–24	4.5	0–11	15	6–44
Problems in communication								
No	7	0–26	2	0–16	4	0–16	25	7–74.5
Yes	2	0–29	0	0–5.5	7	0–16	21.5	7–60
Symptoms								
Headache	9	2–45	6	0–30	2	0–11	30	11–86
Headache only	10	4–101	17.5	5–64	2	0–10	61	20–197
Headache plus	6	2–18.5	4	0–24	2	0–15	23	7–60
Behavioural/cognitive	14	3–62	4	0–16	9	0–19	39	13–90
Confusion	16.5	7–31	1.5	0–6	2.5	0–16	18.5	4.5–41
Memory	14	2–62	5	0–21	11	2–21	62	35–95
Focal neurology	5	0–14	0	0–7.5	9	0–24	21	7–61
Fits, faints or falls	3.5	0–30	0	0–5	0	0–11	10	0–42
Non-specific neurological	12.5	0–28	15	0–35	8	1–17	50	43–65
Other/non-specific	3	0–22	3	0–80	4.5	0–7	16	7–66
Investigation before referral								
No	5	0–18	0	0–4	3.5	0–15	14.5	5–50
Yes	13	1–31	11	4–43	7	0–19	55.5	30–110
Type of referral								
Emergency	4.5	0–18	0	0–6	0	0–3	14	6–39
Not referred by practice	10	0–22	2	0–6.5	0	0–7	7	0–23
2 week/private	13.5	5–30.5	4	0–16	8	5–11.5	39	15–78
Routine	6	0–33	7	0–80	24.5	12.5–53.5	81	50–141
Which specialist to be referred								
A&E	5	0–10	0	0–4	0	0–7	11	7–25
Neurosur/Neurol	8.5	1–26	5	0–33	8	1–19	43.5	10–83
Med/stroke/opth/paeds/miscl	5	0–29	1	0–11	3	0–19	27	8–82

A&E, accident and emergency; Neurosur/neurol, neurosurgery and neurology; Med/stroke/opth/paeds/miscl, medicine and geriatrics, stroke, ophthalmology, paediatrics and miscellaneous.

^a^Patient with missing values (*n* = 28) are excluded.

^b^Patient with missing values (*n* = 45) are excluded.

^c^Patient with missing values (*n* = 49) are excluded.

^d^Patient with missing values (*n* = 46) are excluded.

Table 3 shows the ORs and 95% CI for the longest quartile of time intervals for each stage of the patient pathway. Compared to ‘fits, faints or falls’, headache and the non-specific neurological groups showed a significantly elevated OR for the referral (OR = 6.47, 95% CI = 1.22, 34.3 and OR = 11.9, 95% CI = 1.82, 77.8, respectively). When we looked at the subgroups, headache only (i.e. headache without any other reported features) and memory only, they showed larger ORs for the total pathway interval (OR = 4.11, 95% CI = 1.10, 15.5 and OR = 4.82, 95% CI = 1.15, 20.1, respectively), which was mainly driven by the slower primary care interval (OR = 11.8, 95% CI = 1.88, 73.9 and OR = 10.9, 95% CI = 1.79, 66.1, respectively). GP diagnostic investigations before referral were also associated with slower referral and slower overall pathways. Unsurprisingly, patients who were referred routinely had longer primary care and specialist delays, with referral to accident and emergency having shorter patient, specialist and pathway interval. The results of non-imputed model are shown in supplementary table 2.

**Table 3.** Odds ratios (OR) and 95% confidence intervals (CI) for slowest quartile time along patient pathway to diagnosis

	Time to diagnosis^†^											
	Slowest quartile for first symptom to first attend the GPs			Slowest quartile for first attend the GPs to referral			Slowest quartile for referral to see the specialist			Slowest quartile for first symptoms to see the specialist		
	*n*/total	OR	95% CI	*n*/total	OR	95% CI	*n*/total	OR	95% CI	*n*/total	OR	95% CI
Age group^a^												
<60	21/84	1.01	0.43–2.35	24/84	2.53	0.98–6.52	20/84	1.74	0.62–4.86	25/84	1.38	0.60–3.19
60–69	14/56	1.00	(reference)	9/56	1.00	(reference)	9/56	1.00	(reference)	14/56	1.00	(reference)
>60	19/86	0.83	0.36–1.91	20/86	1.72	0.66–4.51	26/86	2.46	0.99–6.17	19/86	0.88	0.27–2.12
Sex^b^												
Female	24/105	1.00	(reference)	25/105	1.00	(reference)	25/105	1.00	(reference)	25/105	1.00	(reference)
Male	30/121	1.08	0.54–2.14	28/121	0.96	0.49–1.89	30/121	1.18	0.59–2.35	33/121	1.13	0.60–2.10
Ethnicity^c^												
White British	48/195	1.00	(reference)	48/195	1.00	(reference)	46/195	1.00	(reference)	52/195	1.00	(reference)
Other	7/31	0.92	0.34–2.51	5/31	0.43	0.13–1.40	9/31	1.23	0.47–3.17	6/31	0.61	0.21–1.78
Housebound^d^												
No	54/196	1.00	(reference)	42/196	1.00	(reference)	48/196	1.00	(reference)	54/196	1.00	(reference)
Yes	1/31	0.10	0.01–0.77	11/31	2.45	0.90–6.69	7/31	0.61	0.18–2.03	4/31	0.42	0.11–1.60
Problems in communication^d^												
No	48/196	1.00	(reference)	49/196	1.00	(reference)	48/196	1.00	(reference)	52/196	1.00	(reference)
Yes	7/30	0.93	0.32–2.72	4/30	0.44	0.12–1.65	7/30	0.80	0.27–2.37	6/30	0.78	0.25–2.41
Symptoms^d^												
Headache	13/47	1.13	0.39–3.22	15/47	6.47	1.22–34.3	10/47	1.18	0.39–3.61	15/47	2.33	0.80–6.80
Headache only	6/16	1.96	0.54–7.05	8/16	11.8	1.88–73.9	3/16	0.92	0.18–4.55	7/16	4.11	1.10–15.5
Headache plus	7/31	0.81	0.23–2.80	8/31	4.54	0.78–26.5	7/31	1.34	0.39–4.54	8/31	1.68	0.50–5.60
Behavioural/cognitive	10/28	1.64	0.55–4.85	7/28	5.41	0.98–29.8	8/28	1.59	0.47–5.37	9/28	2.62	0.77–8.90
Confusion	3/14	0.97	0.22–4.28	1/14	1.55	0.11–21.3	3/14	1.06	0.17–6.53	2/14	1.13	0.20–6.59
Memory	6/14	2.55	0.67–9.68	6/14	10.9	1.79–66.1	5/14	2.15	0.52–8.84	7/14	4.82	1.15–20.1
Focal neurology	14/75	0.70	0.26–1.87	14/75	3.37	0.69–16.52	22/75	1.61	0.60–4.31	18/75	1.79	0.62–5.17
Fits, faints or falls	11/47	1.00	(reference)	3/47	1.00	(reference)	9/47	1.00	(reference)	7/47	1.00	(reference)
Non-specific neurological	3/11	0.96	0.16–5.79	5/11	11.9	1.82–77.8	3/11	1.73	0.36–8.45	2/11	1.29	1.96–8.51
Other/non-specific	5/18	1.15	0.27–4.88	7/18	8.23	1.03–66.0	3/18	0.71	0.14–3.60	7/18	3.35	0.89–12.65
Investigation before referral^d^												
No	31/153	1.00	(reference)	20/153	1.00	(reference)	34/153	1.00	(reference)	24/153	1.00	(reference)
Yes	24/73	2.02	1.01–4.03	32/73	5.53	2.62–11.67	21/73	1.33	0.68–2.60	34/73	4.81	2.36–9.79
Type of referral^d^												
Emergency	20/98	1.00	(reference)	16/98	1.00	(reference)	11/98	1.00	(reference)	17/98	1.00	(reference)
Not referred by practice	7/38	0.79	0.24–2.55	5/38	0.70	0.18–2.73	6/38	1.57	0.34–7.22	4/38	0.57	0.17–1.89
2 week/private	13/46	1.56	0.67–3.61	14/46	2.26	0.89–2.73	10/46	2.17	0.74–6.39	13/46	1.78	0.72–4.44
Routine	15/44	2.08	0.86–4.99	18/44	3.67	1.53–8.85	28/44	14.5	5.13–40.8	24/44	5.72	2.26–14.5
Which specialist to be referred^d^												
A&E	3/24	1.00	(reference)	3/24	1.00	(reference)	2/24	1.00	(reference)	1/24	1.00	(reference)
Neurosur/neurol	20/84	2.81	0.53–15.0	28/84	4.35	0.68–27.8	22/84	5.60	0.69–45.6	23/84	7.42	0.83–66.2
Med/stroke/opth/paeds/ miscl	33/118	3.82	0.73–19.9	21/118	1.97	0.32–11.9	31/118	5.52	0.69–44.1	34/118	9.45	1.03–87.1

A&E, accident and emergency; Neurosur/neurol, neurosurgery and neurology; Med/stroke/opth/paeds/miscl, medicine and geriatrics, stroke, ophthalmology, paediatrics and miscellaneous.

^a^Adjusted for sex and ethnicity; ^b^Adjusted for age group and ethnicity; ^c^Adjusted for age group and sex; ^d^Adjusted for age group, sex and ethnicity.

^†^Long time to diagnosis defined as worst quartile of time to diagnosis period.

Both headache and behavioural/cognitive changes and non-specific symptoms were associated with at least three or more presentations before referral (Table 4) and this was most marked for headache only (OR = 7.92, 95% CI = 1.80, 34.8) and memory complaints (OR = 6.09, 95% CI = 1.30, 28.6). GPs considered that faster access to investigations would have helped for both headaches and focal neurology symptoms. GPs retrospectively reported that there had been avoidable delays for patients presenting with headache only in the patient journey (OR = 3.64, 95% CI = 0.83, 15.9) but this was consistent with chance.

**Table 4.** The association between symptom domain and frequent attendance, GP perception of need for rapid access investigations and avoidable delay

	*n*/total	Attend 3+ times	95% CI		Rapid access investigations			Avoidable delays	
		OR		*n*/total	OR	95% CI	*n*/total	OR	95% CI
Symptoms^a^									
Headache	21/44	4.50	1.39–14.6	18/45	7.27	1.83–28.9	13/46	2.63	0.81–8.59
Headache only	11/16	7.92	1.80–34.8	11/15	42.77	7.01–261.2	5/16	3.64	0.83–15.9
Headache plus	10/28	3.27	0.91–11.8	7/30	2.88	0.62–13.4	8/30	2.17	0.59–8.04
Behavioural/cognitive	11/25	4.32	1.18–15.8	5/25	2.02	0.40–10.2	3/27	1.00	0.21–4.67
Confusion	4/12	2.99	0.61–14.8	0/12	1.00		2/14	1.38	0.22–8.47
Memory	7/13	6.09	1.30–28.6	5/13	4.13	0.74–23.1	1/13	0.64	0.07–6.29
Focal neurology	22/64	2.71	0.88–8.34	17/68	4.30	1.14–14.1	16/74	2.13	0.70–6.45
Fits, faints or falls	7/36	1.00	(reference)	4/40	1.00	(reference)	6/46	1.00	(reference)
Non-specific neurological	5/11	4.12	0.79–21.4	1/11	1.27	0.11–14.1	3/11	3.59	0.65–19.66
Other/non-specific	10/15	10.17	2.12–48.8	1/14	0.91	0.08–9.86	2/17	0.83	0.14–6.94

^a^ORs adjusted for age group, gender and ethnicity.

## Discussion

This is the first study to examine how different symptoms affect the patient pathway interval, using a representative sample of brain tumour cases from the NACDPC study. We find marked variability in time from symptom onset to first specialist attendance for patients with brain tumours, depending on their symptoms. Overall, the median time from symptom presentation until being seen by a specialist is <4 weeks. Patients presenting with headaches, behavioural/cognitive changes or other/non-specific symptoms attended their GP more frequently before referral; headache only and memory loss are associated with a much slower patient pathway mainly due to delays in referral to a specialist (secondary care). In addition, younger patients under the age of 60 years and patients over the age 69 also tend to experience delays in referral and specialist consultation.

Most previous studies of the diagnostic pathway have focused on very specific tumour types, e.g. vestibular schwannoma (20), intradural spinal cord tumours (21), pituitary adenomas (21), acoustic neuromas (22), central nervous lymphomas (23) or intracranial germ cell tumours (24) (e.g. 23,25,26). Similarly, non-specific or more subtle features such as personality changes were associated with delayed referral in a case series of 58 patients with primary central nervous system lymphoma (23,27). Retrospective interviews with patients and relatives can elicit prior histories of more subtle problems such as cognitive or personality change, though these symptoms may be ignored by the patient (9).

The positive predictive value of headache for adult patients with brain tumours is low (0.09% overall but 0.12% in 60- to 69-year-olds) as compared to new-onset seizure (1.2%) (16). Since headache is a common complaint, it is difficult for GPs and other doctors to differentiate less serious causes of headache from headaches secondary to a brain tumour. Headaches associated with brain tumours are frequently of ‘tension’ type or mimic migraine (8) and the best clues are increasing frequency and severity and headache features (e.g. worsening with cough or bending, nocturnal headaches or headaches on wakening). The development of additional symptoms e.g. focal neurology or signs (papilloedema) will strongly support the diagnosis. This underlines the importance that GPs search for the presence of additional symptoms, such as behavioural/cognitive changes if uncertain as to whether a patient with headache requires investigations or specialist referral. The use of simple cognitive screening tests, such as semantic verbal fluency, may help. This requires assessment of how many animals the patient can name in one minute and has been previously demonstrated to be worse in brain tumour patients whose initial presenting symptom was headache/headache ‘plus’ (28,29).

### Strengths and limitations

The study has good generalizability to other high-income healthcare settings, as cases were identified consecutively from primary care, without any selection by specialist units. Most studies do not prospectively collect data on patient delay, so cannot untangle the patient pathway into all its constituent components. However, we were forced to group various symptoms into domains to achieve sufficient power due to the sample size. In addition, the reporting of potentially avoidable delays and whether further investigation would have helped was done retrospectively by the GPs, so may have been biased by the actual patient outcomes. For some non-acute features, such as behavioural change, patients may have incorrectly reported the date of symptom onset. Some patients who had a first-ever presentation directly to accident and emergency departments and were hospitalised would have not been included in this dataset, although this is not directly relevant to the issue of improving diagnostic delay in elective primary care. We included headaches associated with ‘nausea’ and ‘vomiting’ (N&V) under the headache plus group given the lack of qualifying information in the data available. Ideally, though there would be a distinction between N&V seen in common conditions such as migraine and ‘atypical’ or ‘red flag’ N&V (such as N&V confined to early mornings, or on bending down) which alerts the GP to the possibility of more serious pathology—such as a brain tumour. We could not look at how presentation and delay were associated with type of brain tumour as we did not have data on the specific pathology, size and location. This would be of interest as it would also be associated with management and prognosis.

Interestingly, GPs considered that more rapid access to investigations, such as neuroimaging, would have helped, particularly for less specific symptoms such as headache (30,31). This important question needs to be looked at in terms of cost-effectiveness given the potential large number of patients that will turn out to have a normal scan. Current National Institute for Health and Care Excellence (NICE) guidance states ‘Consider an urgent direct access MRI scan of the brain (or CT scan if MRI is contraindicated) (to be performed within 2 weeks) to assess for brain or central nervous system cancer in adults with progressive, sub-acute loss of central neurological function.’ (32) Patients with only headache or simple memory loss would not in themselves be considered to meet these criteria. In addition, there is an implicit assumption that the reduction in the diagnostic interval for patients presenting with headaches and memory loss would translate into better clinical outcomes, which may or may not be true. Future work should examine whether geographical areas with rapid access to neuroimaging have reduced delay in time to diagnosis and whether this translates to differences in patient management, morbidity and survival.

## Conclusions

Whilst many patients with brain tumours are diagnosed rapidly, GPs and other doctors currently face a diagnostic challenge when deciding whether to refer patients with headaches and memory complaints. Future work needs to identify whether any additional features or other simple inexpensive tests could be administered in primary care that could help reduce the time to diagnosis in these patients.

## Supplementary Material

Supplementary data are available at *Family Practice* online.

Supplementary Material

## Declaration

Funding: This project has been funded by a grant from the Brain Tumour Charity (GN-000295) as part of a programme of work on understanding the diagnostic pathway for brain tumours in adults and its potential impact on clinical care and outcomes. WH is the joint PI of the multi-institutional ‘CanTest’ Research Collaborative funded by a Cancer Research UK Population Research Catalyst award (C8640/A23385).

Ethical approval: NACDPC was set up as an audit exercise. Participating cancer networks were required to gain local approval for this audit. No patient identifiable data were collected. All data submitted to the National Cancer Intelligence Network (NCIN) for analysis were held on the same IT system and under the same information governance arrangements as apply to cancer registries.

Author contributions: All the authors were involved in the conception of the study. YBS and MO undertook the data analysis. KZ, PB and RG helped devise the symptom domains. All the authors helped with the data interpretation. MO and YBS co-wrote the first draft of the paper which was then modified after comments and suggestions from the other authors.Disclosures: The authors have no financial disclosures. Willie Hamilton is an associate editor of *Family Practice*.
