# Supplementary material for: Symptoms in primary care with time to diagnosis of brain tumours
Source: Fam Pract. 2018 Feb 6;35(5):551–8. doi: 10.1093/fampra/cmx139 (PMC6142711; doi:10.1093/fampra/cmx139)
Supplement: Supplementary Material [file cmx139_suppl_supplementary-material.docx]

| Supplementary table 1. Symptom domains with some examples of specific symptoms | |
| --- | --- |
| 1. Headache |  |
| 1-1. Headache only | Headache |
| 1-2. Headache plus | Headache plus any other neurological feature |
| 2. Behavioural / cognitive | |
| 2-1. Confusion | Confusion |
| 2-2. memory | e.g. Memory Loss |
|  | e.g. Poor concentration |
|  | e.g. Cognitive decline |
|  | e.g. strange behaviour |
| 3. Focal neurology | Hemiparesis |
|  | Ataxia |
|  | TIA, CVA, Stroke, stroke-like symptoms |
|  | Incoordination |
|  | Dysphasia |
|  | Double vision, diplopia, loss of vision |
|  | Slurred speech |
|  | Vertigo |
|  | Sudden onset deafness |
|  | 6th nerve palsy |
|  | Weakness and numbness |
|  | Poor co-ordination |
|  | Squint |
| 4. Fits, faints or falls | Seizure |
|  | Collapse |
|  | Fit |
|  | Funny turns |
|  | Fainting |
|  | Falls |
|  | Convulsion |
|  | Strange sensation in stomach and strange taste sensation |
| 5. Non-specific neurological | Poor balance |
|  | Dizziness |
|  | Gait abnormality |
| 6. Other/non-specific | Vomiting |
|  | Nausea |
|  | Breast Lump |
|  | Lethargy |
|  | Sweating |
|  | General malaise |
|  | UTI |
|  | Tinea leg |
|  | Cyst |
|  | Not known |
|  |  |

| Supplementary table 2. Odds ratios and 95 confidence intervals for long time to diagnosis vs. short time to diagnosis without imputation | | | | | | | | | | | | | | | |
| --- | --- | --- | --- | --- | --- | --- | --- | --- | --- | --- | --- | --- | --- | --- | --- |
|  | Time to diagnosis | | | | | | | | | | | | | | |
|  | Slowest quartile for first symptom to first attend the GPs | | |  | Slowest quartile for first attend the GPs to Referral | | |  | Slowest quartile for referral to see the specialist | | |  | Slowest quartile for first symptoms to see the specialist | | |
|  | n/total | Odds ratio | 95%CI |  | n/total | Odds ratio | 95%CI |  | n/total | Odds ratio | 95%CI |  | n/total | Odds ratio | 95%CI |
| **Age group^*1^** |  |  |  |  |  |  |  |  |  |  |  |  |  |  |  |
| <60 | 17/68 | 0.87 | 0.35-2.15 |  | 21/64 | 3.99 | 1.33-11.9 |  | 17/63 | 3.86 | 1.17-12.7 |  | 22/74 | 1.47 | 0.61-3.58 |
| 60-69 | 12/46 | 1.00 |  |  | 7/46 | 1.00 |  |  | 7/48 | 1.00 |  |  | 11/48 | 1.00 |  |
| >60 | 14/64 | 0.69 | 0.26-1.82 |  | 16/63 | 2.78 | 0.90-8.56 |  | 21/65 | 5.05 | 1.54-16.6 |  | 15/73 | 0.88 | 0.34-2.28 |
|  |  |  |  |  |  |  |  |  |  |  |  |  |  |  |  |
| **Sex^*2^** |  |  |  |  |  |  |  |  |  |  |  |  |  |  |  |
| Female | 19/81 | 1.00 |  |  | 20/84 | 1.00 |  |  | 21/86 | 1.00 |  |  | 22/92 | 1.00 |  |
| Male | 26/100 | 1.19 | 0.56-2.52 |  | 24/93 | 1.16 | 0.55-2.46 |  | 24/94 | 1.57 | 0.74-3.32 |  | 27/106 | 1.24 | 0.61-2.51 |
|  |  |  |  |  |  |  |  |  |  |  |  |  |  |  |  |
| **Ethnicity^*3^** |  |  |  |  |  |  |  |  |  |  |  |  |  |  |  |
| White british | 34/142 | 1.00 |  |  | 36/139 | 1.00 |  |  | 33/141 | 1.00 |  |  | 40/158 | 1.00 |  |
| Other | 6/24 | 1.14 | 0.41-3.17 |  | 4/25 | 0.42 | 0.13-1.35 |  | 7/25 | 1.08 | 0.40-2.90 |  | 5/25 | 0.69 | 0.24-2.00 |
|  |  |  |  |  |  |  |  |  |  |  |  |  |  |  |  |
| **Housebound^*4^** |  |  |  |  |  |  |  |  |  |  |  |  |  |  |  |
| No | 42/153 | 1.00 |  |  | 35/153 | 1.00 |  |  | 40/155 | 1.00 |  |  | 44/165 | 1.00 |  |
| Yes | 1/19 | 0.16 | 0.20-1.32 |  | 7/19 | 2.41 | 0.81-7.14 |  | 4/20 | 0.37 | 0.10-1.45 |  | 3/23 | 0.48 | 0.13-1.80 |
|  |  |  |  |  |  |  |  |  |  |  |  |  |  |  |  |
| **Problems in communication^*4^** | | | | |  |  |  |  |  |  |  |  |  |  |  |
| No | 40/154 | 1.00 |  |  | 39/151 | 1.00 |  |  | 38/153 | 1.00 |  |  | 44/168 | 1.00 |  |
| Yes | 5/19 | 1.3 | 0.42-4.03 |  | 3/20 | 0.55 | 0.14-2.09 |  | 5/21 | 0.75 | 0.23-2.37 |  | 5/22 | 1.03 | 0.35-3.08 |
|  |  |  |  |  |  |  |  |  |  |  |  |  |  |  |  |
| **Symptoms^*4^** |  |  |  |  |  |  |  |  |  |  |  |  |  |  |  |
| Headache | 12/43 | 0.83 | 0.25-2.73 |  | 14/41 | 12.48 | 1.46-106.6 |  | 8/41 | 1.09 | 0.29-4.10 |  | 14/45 | 1.79 | 0.57-1.79 |
| Headache only | 6/15 | 1.43 | 0.32-6.30 |  | 7/14 | 19.02 | 1.87-193.6 |  | 2/14 | 0.76 | 0.12-4.90 |  | 7/15 | 3.12 | 0.73-13.2 |
| Headache plus | 6/28 | 0.58 | 0.14-2.39 |  | 7/27 | 9.43 | 1.03-86.7 |  | 6/27 | 1.28 | 0.31-5.30 |  | 7/30 | 1.32 | 0.36-4.82 |
| Behavioural/cognitive | 9/23 | 2.26 | 0.66-7.80 |  | 7/23 | 9.30 | 0.98-87.8 |  | 7/23 | 1.50 | 0.37-6.11 |  | 8/25 | 2.66 | 0.77-9.21 |
| Confusion | 3/10 | 1.44 | 0.26-8.04 |  | 1/10 | 3.70 | 0.20-69.9 |  | 2/10 | 1.61 | 0.23-11.3 |  | 2/12 | 1.25 | 0.21-7.60 |
| Memory | 6/13 | 2.99 | 0.71-12.7 |  | 6/13 | 13.84 | 1.33-143.9 |  | 5/13 | 1.44 | 0.29-7.08 |  | 6/13 | 4.36 | 1.03-18.4 |
| Focal neurology | 10/61 | 0.64 | 0.21-2.00 |  | 12/64 | 6.04 | 0.72-50.7 |  | 20/65 | 1.63 | 0.50-5.29 |  | 15/65 | 1.59 | 0.53-4.71 |
| Fits, faints or falls | 9/34 | 1.00 | (reference) |  | 2/29 | 1.00 | (reference) |  | 6/31 | 1.00 | (reference) |  | 6/41 | 1.00 | (reference) |
| Cranial nerve | 2/8 | 1.35 | 0.21-8.71 |  | 5/10 | 20.22 | 1.71-239.0 |  | 3/10 | 2.30 | 0.38-14.0 |  | 2/9 | 1.57 | 0.25-9.91 |
| Other/non-specific | 3/12 | 1.27 | 0.24-6.59 |  | 4/10 | 13.37 | 1.19-149.6 |  | 1/10 | 0.36 | 0.04-3.65 |  | 4/13 | 2.72 | 0.58-12.8 |
|  |  |  |  |  |  |  |  |  |  |  |  |  |  |  |  |
| **Investigation before referral^*4^** | | |  |  |  |  |  |  |  |  |  |  |  |  |  |
| No | 23/114 | 1.00 |  |  | 14/110 | 1.00 |  |  | 27/114 | 1.00 |  |  | 20/130 | 1.00 |  |
| Yes | 21/64 | 1.85 | 0.87-3.94 |  | 30/65 | 6.96 | 3.04-15.9 |  | 18/63 | 1.20 | 0.56-2.59 |  | 29/62 | 5.14 | 2.46-10.8 |
|  |  |  |  |  |  |  |  |  |  |  |  |  |  |  |  |
| **Type of referral^*4^** | |  |  |  |  |  |  |  |  |  |  |  |  |  |  |
| Emergency | 17/80 | 1.00 |  |  | 13/82 | 1.00 |  |  | 9/83 | 1.00 |  |  | 14/82 | 1.00 |  |
| Not referred by practice | 3/15 | 0.98 | 0.24-4.02 |  | 1/8 | 0.89 | 0.09-8.43 |  | 1/11 | 1.50 | 0.16-14.3 |  | 2/27 | 0.33 | 0.07-1.60 |
| 2 week/private | 12/40 | 1.66 | 0.65-4.27 |  | 12/41 | 2.21 | 0.82-5.97 |  | 7/40 | 2.47 | 0.78-7.81 |  | 11/39 | 2.04 | 0.77-5.37 |
| Routine | 10/31 | 2.11 | 0.78-5.74 |  | 15/37 | 3.58 | 1.38-9.27 |  | 22/36 | 15.74 | 5.25-47.2 |  | 17/31 | 6.94 | 2.56-18.8 |
|  |  |  |  |  |  |  |  |  |  |  |  |  |  |  |  |
| **Which specialist to be referrerd^*4^** | | | | | | | | | | | | | | | |
| Med/Eyes/Stroke/Miscell | 2/16 | 1.00 |  |  | 2/15 | 1.00 |  |  | 1/16 | 1.00 |  |  | 1/17 | 1.00 |  |
| Neurosur/Neurol/Paeds | 15/58 | 3.82 | 0.45-32.5 |  | 23/59 | 4.56 | 0.90-23.2 |  | 18/62 | 6.50 | 0.77-55.0 |  | 19/64 | 6.08 | 0.73-50.7 |
| A&E | 25/95 | 6.00 | 0.73-49.4 |  | 19/99 | 1.86 | 0.37-9.33 |  | 25/98 | 5.57 | 0.67-46.1 |  | 27/95 | 8.18 | 0.99-67.7 |
| ^ᵻ^Long time to diagnosis defined as worst quartile of time to diagnosis period | | | | | | | | | | | | | | | |
| ^*1^Adjusted for sex and ethnicity; ^*2^Adjusted for age group and ethnicity; ^*3^Adjusted for age group and sex; ^*4^Adjusted for age group, sex, and ethnicity | | | | | | | | | | | | | | | |
| ^†1^Accident & Emergency, ^†2^ Neurosurgery & Neurology, ^†3^ Medicine & Geriatrics, stroke, Ophthalmology, Paediatrics & Miscellaneous. | | | | | | | | | | | | | | | |
